# Measuring tumor depth of Bowen's disease by optical coherence tomography

**DOI:** 10.1111/srt.13282

**Published:** 2023-02-02

**Authors:** Shima Ahmady, Tom Wolswijk, Patricia Joan Nelemans, Fieke Adan, Astrid Ingrid Paulien Vernemmen, Véronique Winnepenninckx, Nicole Wilhelmina Johanna Kelleners‐Smeets, Klara Mosterd

**Affiliations:** ^1^ Department of Dermatology Maastricht University Medical Center+ Maastricht The Netherlands; ^2^ GROW Research Institute for Oncology and Reproduction Maastricht University Maastricht The Netherlands; ^3^ Department of Epidemiology Maastricht University Maastricht The Netherlands; ^4^ Department of Pathology Maastricht University Medical Center+ Maastricht The Netherlands

1

Bowen's disease (BD) is often described as squamous cell carcinoma in situ, because atypical keratinocytes in BD are confined to the epidermis.^1^ Histopathologically, there is full thickness cytonuclear atypia and acanthosis with thickened and elongated rete ridges which makes tumor depth (TD) variable among cases.^2^ TD may be of clinical importance because it has been proposed that TD may influence the effectiveness of photodynamic therapy (PDT).^3^, ^4^, ^5^, ^6^

BD is commonly diagnosed by biopsy and subsequent histopathological examination of the obtained specimen which also enables the measurement of the TD. However, because non‐invasive therapy is suitable for BD, a non‐invasive diagnostic procedure may be preferable. It is therefore of interest to evaluate whether measuring TD non‐invasively leads to accurate results.

Optical coherence tomography (OCT) is a safe non‐invasive tool that generates real‐time in vivo cross‐sectional images of the skin. Tumor characteristics for BD have been previously described and have similarities with histopathology.^7^ It is possible to measure epidermal thickness on OCT, but it has never been investigated whether it can be used to measure TD in BD lesions. We therefore investigated whether TD of BD lesions can be accurately measured on OCT by assessing the extent of agreement between OCT and histopathological measurements.

In this cohort study, patients underwent an OCT scan (Vivosight Multi‐beam Swept‐Source Frequency Domain OCT) prior to punch biopsy. Biopsy specimens were obtained conform regular care. A dermatopathologist, blinded to the TD on OCT, measured TD on histopathology by drawing a perpendicular line from the deepest part of the tumor toward the stratum granulosum. If an adnexal extension of BD grew deeper than other parts of the tumor, the depth of adnexal extension was included in the estimate of TD. A trained OCT assessor, blinded to the histopathological TD, measured TD on OCT by drawing a perpendicular line from the deepest visible part of the epidermis, toward the stratum granulosum. If keratosis made the dermo‐epidermal junction invisible throughout the scan, the lesion was excluded. TD as measured by histopathology and OCT was expressed in millimeters.

A total of 42 patients (mean age 76) with histopathologically verified BD lesions were included in this study. The mean TD according to histopathology and OCT was 0.60 mm (SD ± 0.39) and 0.47 mm (SD ± 0.16), respectively. A Bland–Altman plot was constructed to visualize the extent of agreement between OCT and histopathological measurements (Figure 1). The *Y*‐axis represents the difference between the two measurements (TD OCT minus TD histopathology), whereas the *X*‐axis represents the mean of both measurements and serves as an estimated measure of TD. The plot shows that the mean difference was −0.13 mm with SD ± 0.44, indicating that there was a systematic bias toward slight underestimation of histopathological TD by OCT. The 95% limits of agreement are situated between −0.99 mm and +0.72 mm, which implies that discrepancies between histopathology and OCT can be substantial. Additionally, it can be observed that the TD of thinner tumors tends to be overestimated by OCT, whereas TD of thicker tumors tends to be underestimated by OCT. Underestimation of TD by OCT was significantly more pronounced in tumors with adnexal extension (mean discrepancy: −0.51 mm (SD ± 0.49), compared to tumors without adnexal extension (mean discrepancy: +0.02 mm (SD ± 0.31; *P* = 0.0001).

**FIGURE 1:**
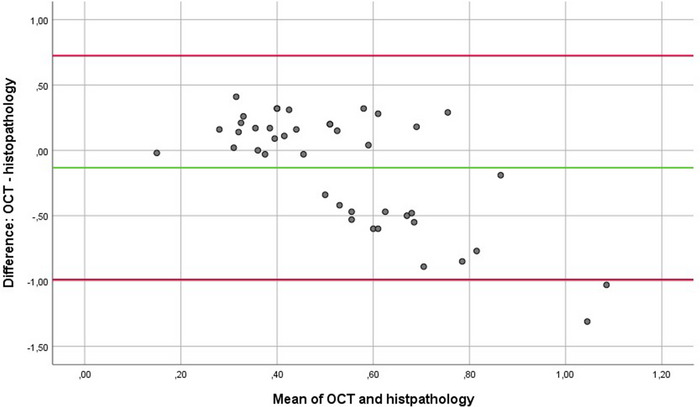
Bland–Altman plot of all lesions plotting the difference between OCT and histopathology (TD OCT minus TD histopathology) against the mean of OCT and histopathology which serves as an estimated measure of TD. Green line indicates the mean difference. Red lines indicate the upper and lower limits of agreement. Abbreviations: OCT, optical coherence tomography; TD, tumor depth

This explorative study showed that there is a low level of agreement between histopathology and OCT for determining TD in BD lesions. Thin tumors tend to be overestimated by OCT, whereas thick lesions tend to be underestimated by OCT. Various explanations can be given for these findings.

The first explanation may be that pathologists evaluate biopsy specimens on a cellular level, whereas the conventional OCT only provides information on the gross architecture of the skin. The greatest distance between stratum basale and stratum granulosum on OCT may not consist of atypical keratinocytes; a requisite for diagnosing BD. Therefore, the thickest part of the epidermis may not be considered BD by histopathology, causing an overestimation of TD by OCT, explaining why especially thinner tumors were overestimated by OCT. With line‐field confocal optical coherence tomography (LC‐OCT) and high‐definition optical coherence tomography (HD‐OCT), it is possible to visualize cellular atypia but clinical usefulness may still be limited for measuring TD because penetration depth is 500 and 540 μm, respectively.^8^, ^9^, ^10^

A second explanation for the low agreement between OCT and histology could be that the diameter of biopsy is limited to 3 mm, whereas OCT covers an area of 6 mm. As a result, OCT may include a thicker part of the tumor outside of the biopsied area resulting in a higher estimate of TD than when measured by histopathological examination.

The last explanation for disagreement is tumoral extension along adnexal extensions. Adnexal extension is easily identified in histopathologic specimens by the presence of atypical keratinocytes along the adnexal structure. However, because OCT does not provide information on a cellular level it is impossible to detect tumoral extension along adnexal structures on OCT. The depth of the adnexal extension may surpass TD in the remainder of the tumor causing underestimation by OCT.

In conclusion, TD measurement of BD by conventional OCT is not representative for TD measurement on histopathological slides of biopsy specimens because the extent of agreement on TD between OCT and histopathology is low. TD in thinner tumors tends to be overestimated by OCT, whereas TD of thicker tumors tends to be underestimated.

## CONFLICT OF INTEREST

The authors declare that there is no conflict of interest that could be perceived as prejudicing the impartiality of the research reported.

## Data Availability

Data are available upon request.
